# Etymologia: *Bacillus anthracis*

**DOI:** 10.3201/eid2009.ET2009

**Published:** 2014-09

**Keywords:** etymologia, Bacillus anthracis, bacteria, anthrax, cutaneous anthrax, Robert Koch

## *Bacillus anthracis* [bə-silʹəs an-thraʹsis]

A large, gram-positive, rod (bacillus), *Bacillus anthracis* is the causative agent of anthrax (Greek for “coal”), named for the black lesions of cutaneous anthrax. In 1850, Rayer and Davaine discovered the rods in the blood of anthrax-infected sheep, setting the stage for Koch to link the disease to the bacterium in 1876, after he performed a series of experiments that fulfilled what came to be known as Koch’s postulates. This was among the first times a microorganism was conclusively linked with a specific disease.
